# Helicopter emergency medical services demonstrate reduced time to emergency anaesthesia in an undifferentiated trauma population: a retrospective observational analysis across three major trauma networks

**DOI:** 10.1186/s13049-024-01313-y

**Published:** 2024-12-27

**Authors:** Daniel Heritage, Joanne Griggs, Jack Barrett, Scott Clarke, Rory Carroll, Richard Lyon, Duncan Bootland

**Affiliations:** 1 Air Ambulance Charity Kent Surrey Sussex, Redhill Aerodrome, Redhill, Surrey RH1 5YP UK; 2 University Hospital Sussex, Brighton and Hove, Brighton UK; 3 Department of Health Sciences, University of Surrey, Guildford, GU2 7XH UK; 4 St George’s Hospital, Tooting, Blackshaw Road, London, SW17 0QT UK; 5 South East Coast Ambulance Foundation Trust, Crawley, UK

**Keywords:** Trauma, Pre-hospital, Anaesthesia, Helicopter emergency medical services

## Abstract

**Background:**

Early rapid sequence induction of anaesthesia (RSI) and tracheal intubation for patients with airway or ventilatory compromise following major trauma is recommended, with guidance suggesting a 45-min timeframe. Whilst on-scene RSI is recommended, the potential time benefit offered by Helicopter Emergency Medical Services (HEMS) has not been studied. We compared the time from 999/112 emergency call to delivery of RSI between patients intubated either in the Emergency Department or pre-hospital by HEMS.

**Methods:**

A retrospective observational cohort study of major trauma patients in South-East England who received a pre-hospital RSI (PHRSI) or Emergency Department RSI (EDRSI) between 2 January 2018 and 24 September 2019. Data were extracted from the UK Trauma Audit and Research Network database. The primary outcome was the time from emergency call to delivery of RSI. Secondary outcomes included mortality at 30-days or hospital discharge, time from arrival of service at hospital or scene to RSI, time from emergency call to Computerised Tomography scan, and conveyance interval. Linear regression was used to model time to RSI in both groups.

**Results:**

Of 378 eligible patients, 209 patients met inclusion criteria. 103 received a PHRSI and 106 received an EDRSI. Most patients were male (n = 171, 82%) and the median age was 48 years (IQR 28–65). 94% sustained a blunt injury mechanism and head was the most injured body region for both cohorts (n = 134, 64%). 63% (n = 67) of patients receiving a PHRSI were conveyed by helicopter. PHRSI was delivered significantly earlier with a median of 64 [IQR 51–75] minutes (95% CI, 60–68) compared with EDRSI with a median of 84 [IQR 68–113] minutes (95% CI, 76–94), *p* < 0.001).

**Conclusion:**

Major trauma patients who had a pre-hospital RSI received this time-critical intervention sooner after their injury than those who received an emergency anaesthetic after conveyance to a specialist hospital. Patient outcome benefit of HEMS delivered early RSI should be explored.

## Background

Major trauma constitutes a significant burden of morbidity and mortality and remains the leading cause of death in those aged under 40, with survivors often suffering life-long disability [1–3]. Major trauma patients can lose the ability to maintain their own airway or ventilate effectively, and the provision of early and effective airway management provided by advanced trauma systems may help to reduce morbidity and mortality after major trauma.

Pre-hospital rapid sequence induction (PHRSI) of anaesthesia and endotracheal intubation augments ventilation and is recommended in patients suffering major trauma who cannot maintain an adequate airway [4, 5, 23]. The National Confidential Enquiry into Patient Outcome and Death (NCEPOD, 2007) [6] concluded that one in eight patients suffering trauma had a partially or completely obstructed airway upon arrival to the hospital [6, 7]. supporting the rationale for the availability of pre-hospital emergency medical (PHEM) teams to deliver pre-hospital RSI at or near the point of injury.

Tracheal intubation outside of the anaesthetic room is associated with higher rates of complications, including lower first-pass success, episodes of hypotension and hypoxia [26]. The National Audit Project 4 (NAP-4) in the United Kingdom showed that at least one in four reported major airway events leading to permanent harm or death occurred in the Intensive Care Unit or Emergency Department [26]. Data surrounding the incidence of adverse airway events after pre-hospital, physician-delivered RSI is sparse. However, the complex and relatively remote pre-hospital environment presents additional challenges to a complex procedure. Implementing standard operating procedures and checklists for Helicopter Emergency medical Services (HEMS) has aimed to reduce human error and make PHRSI as safe as possible [27]. The decision to delay RSI until arrival at the hospital could reduce the number of adverse airway events by virtue of proximity to other senior anaesthetists and airway-trained clinicians.

Although the intervention carries recognised risks, particularly in the critically ill, tracheal intubation reduces the risk of aspiration, which is the leading cause of death associated with airway management [5]. Once established, sedation and controlled ventilation allows the accurate titration of ventilatory parameters to avoid detrimental sequelae in traumatic brain injury (TBI) such as hypoxia or inappropriate changes in intracranial pressure related to hyper- or hypocapnia [8]. However, not all patients with indications for RSI may be attended to by a HEMS team to deliver the intervention. Robust regional data collection is important to determine the proportion of emergency anaesthesia delivered in both the pre-hospital and in-hospital phases of care, to establish whether there is a cohort of patients who would benefit from pre-hospital RSI but for which pre-hospital resources do not currently allow.

The National Institute for Health and Care Excellence (NICE) released a quality statement regarding airway management in the major trauma patient (2018). They recommend rapid sequence induction (RSI) of anaesthesia and endotracheal intubation in the major trauma patient who cannot maintain their own airway and/or ventilation, within 45 min of the initial call to emergency services (NICE, 2018). In the United Kingdom (UK), teams capable of pre-hospital RSI achieve this target in 25% of trauma patients, with a median time of 55-min. [9] Therefore, despite the rationale described above, it may be increasingly time efficient for ground emergency services to convey major trauma patients directly to hospital for an RSI. To our knowledge, exploration of the frequency and timing of pre-hospital RSI (PHRSI) and emergency department (ED) RSI (EDRSI) for trauma patients is unevidenced [10]. We hypothesise that the time from call to emergency services to the delivery of an RSI in major trauma patients is shorter when the RSI is delivered pre-hospital.

This study aims to compare the time from emergency call to delivery of RSI in an undifferentiated major trauma population who require definitive airway management. We will compare time intervals for two cohorts stratified upon location of RSI.

## Methods

### Study setting and design

A retrospective observational cohort study of trauma patients who were conveyed to one of three regional Major Trauma Centres (MTC) and subsequently included in the National Trauma and Audit Research Network (TARN) database. We compared the time from call to emergency services to delivery of RSI for patients attended by Air Ambulance Charity Kent Surrey Sussex (KSS) against those attended by the local ground Emergency Medical Service (EMS) provider alone.

Air Ambulance Charity Kent Surrey Sussex operates a 24/7 helicopter emergency medical service (HEMS) for three counties and three trauma networks in the southeast of England with a resident population of 4.5 million. KSS operates two doctor-paramedic teams simultaneously. Major trauma patients who are conveyed by KSS or ground ambulance will be taken to one of three MTCs: The Royal Sussex County Hospital, Brighton, UK (Sussex Trauma Network), St George’s Hospital, London, UK (Southwest London and Surrey Trauma Network), or King’s College Hospital, London, UK (Southeast London, Kent and Medway Trauma Network). Conveyance to a MTC may involve bypassing Trauma Units.

The HEMS team comprise a specialist Doctor and Paramedic who respond by either helicopter or rapid response vehicle. Doctors have at least 5 years postgraduate experience, including a minimum of six months of in-hospital anaesthesia. Paramedics undergo further specialist training, including theoretical modules on pre-hospital anaesthesia. All clinicians complete an induction and sign-off process prior to independent practice, including both simulation-based education and workplace supervision. Within the three counties over which KSS operates, no other pre-hospital teams provide on-scene RSI. On occasion, a mutual aid request is made for a neighbouring HEMS to perform a critical intervention such as RSI when KSS is otherwise operationally committed.

HEMS are dispatched by dedicated non-clinical dispatchers [24]. Dispatchers interrogate 999/112 calls using a paper-based algorithm with dispatch triggers to determine HEMS activation. ‘Grade 1’ dispatch requires a single trigger, and ‘Grade 2’ dispatch requires two triggers. Dispatch triggers are categorised into three groups: mechanism, condition of the patient and location. Occasionally, HEMS teams can be ‘re-tasked’ on-route for a higher priority mission.

### Study population

We included all adult major trauma patients (≥ 16 years) from the TARN database who received RSI, were conveyed by ground ambulance or KSS to one of three regional MTCs and did not meet any exclusion criteria. The study was undertaken between 2 January 2018, and 24 September 2019.

Study exclusion criteria comprised:

1. Patients < 16 years of age
2. Patients intubated in traumatic cardiac arrest (TCA) without paralysis and sedation
3. Patients who received an RSI but were transferred from another hospital
4. Patients for whom complete data could not be sourced after interrogation of TARN
5. Patients who received an RSI > 60 min following arrival to the ED
6. Patients who were conveyed by KSS but received RSI in the ED
7. Patients who received RSI by neighbouring HEMS service through mutual aid request

Standard TARN patient inclusion criteria comprise: admission for three or more nights, admission to critical care, in-hospital and ED deaths following trauma and transfer to another hospital for specialist care [11]. TARN exclusion criteria comprise: transfer for rehabilitation purposes only, isolated neck of femur or trochanteric fractures in patients aged over 65 years and isolated closed limb fractures (except femoral fractures) [11]. TARN-eligible patients are identified at hospital level by local data coordinators. Patient data are prospectively captured by local coordinators using an electronic system then checked for accuracy and completeness by trained analysts before entering the TARN database [11].

Patients who received EDRSI > 60 min after arrival in ED were excluded because the authors deemed it unlikely that these patients had an immediate requirement for definitive airway management on arrival to ED. This is in-line with existing literature which suggests that RSI performed within 60–120 min after ED arrival generally meets an urgent and not emergency indication [12, 13].

### Primary outcome

The primary outcome was defined as the time (minutes) from emergency 999/112 call to definitive airway management as indicated by RSI time. Analysis compared those who received pre-hospital RSI with those who received RSI in the ED. This outcome was chosen to compare both cohorts against the NICE recommendation of definitive airway management within 45 min of 999/112 emergency call.

### Secondary outcome(s)

Pre-specified secondary outcomes were chosen based upon clinical and operational relevance. Secondary outcomes comprise:

i) Conveyance Interval: As defined by time the conveying resource leaves scene to time of arrival at hospital (min).
ii) Mortality at (30-days) or hospital discharge (y/n).
iii) Arrival of service to RSI: Time taken to deliver RSI (min), defined as the ED arrival time to RSI for EDRSI, and HEMS service arrival time to RSI for PHRSI.
iv) Time from emergency call to Computerised Tomography (CT) scan and time from ED arrival to CT scan.

### Conduct of rapid sequence intubation

PHRSI is delivered by KSS by means of a Standard Operating Procedure (SOP) utilising a combination of Fentanyl, Ketamine and Rocuronium. Indication for PHRSI comprise (1) actual or impending airway compromise, (2) ventilatory failure, (3) unconsciousness, (4) patients who are unmanageable or severely agitated after head injury, (5) anticipated clinical course, whereby a patient is likely to deteriorate on route to hospital, (6) humanitarian need. The process, safety and efficacy of this SOP has been previously published [15]. For EDRSI, the procedure was delivered in-line with standard clinical practice in each MTC. There is no universal SOP for RSI across the three MTCs.

### Ethical considerations

The project was registered with the University of Surrey and met National Institute for Healthcare Research (NIHR, UK) criteria as a service evaluation. All data collected was done so routinely and data protection was assured with a Data Sharing Agreement between TARN and KSS. The project was approved by the KSS Research and Innovation Committee and conducted in accordance with Strengthening the Reporting of Observational Studies in Epidemiology (STROBE) Guidelines [14].

### Data acquisition

Data points were derived from the Trauma Audit and Research Network (TARN) database by an assigned data analyst and were further interrogated by DH and JG. Patient demographics included age, gender, mode of arrival at hospital, mechanism of injury and injury characteristics including initial Glasgow Coma Scale (GCS) score, injury severity score (ISS) and injured body region (denoted by the Abbreviated Injury Scale, (AIS)). Time intervals included the time of emergency call, ground EMS arrival at and departure from scene, patient arrival at hospital, RSI and CT scan. Clinical outcome variables included mortality at 30 days, length of stay in hospital and length of stay on a critical care unit. Data were retrieved from TARN on 1 February 2021, allowing time for follow-up from the receiving hospital to have been entered onto the database.

### Patient and public involvement

Lay representation on the Charity Board at KSS expressed support for research into pre-hospital RSI. Patients were not directly involved in the study design, recruitment, or conduct. Results and clinical interpretation of the study will be shared with both lay representatives and partner organisations as deemed appropriate.

### Statistical analysis

Categorical data are reported as frequency (n) and percent (%) and continuous data as mean and standard deviation (SD) or median and interquartile range (IQR) depending on whether the data were normally distributed or not. One sample Kolmogorov–Smirnov test was used to test data for normality. To explore differences between groups, the Chi Square test was used for categorical data and Mann–Whitney U test was applied to continuous data. Unadjusted and adjusted linear regression models were used to predict time to RSI in each group for the primary outcome measure. For adjusted models the co-variates age (years), ISS and GCS score were included. These were selected through stepwise variable selection, and the model fit was assessed according to its adjusted R squared. The authors acknowledge that ISS would not be available to the HEMS clinician at the time of the incident. However, the authors kept this in the model as a surrogate for injury severity. Missing data were recorded in-line with Strengthening the Reporting of Observational Studies in Epidemiology (STROBE) Guideline [14] and the Reporting of Studies Conducted using Observational Routinely collected Health Data (RECORD) Statement. [15] Statistical significance was set as a two-tailed *p* value < 0.05. Statistical analysis was conducted in R programming (version 4.2.2).

## Results

### Study population

**Fig. 1:**
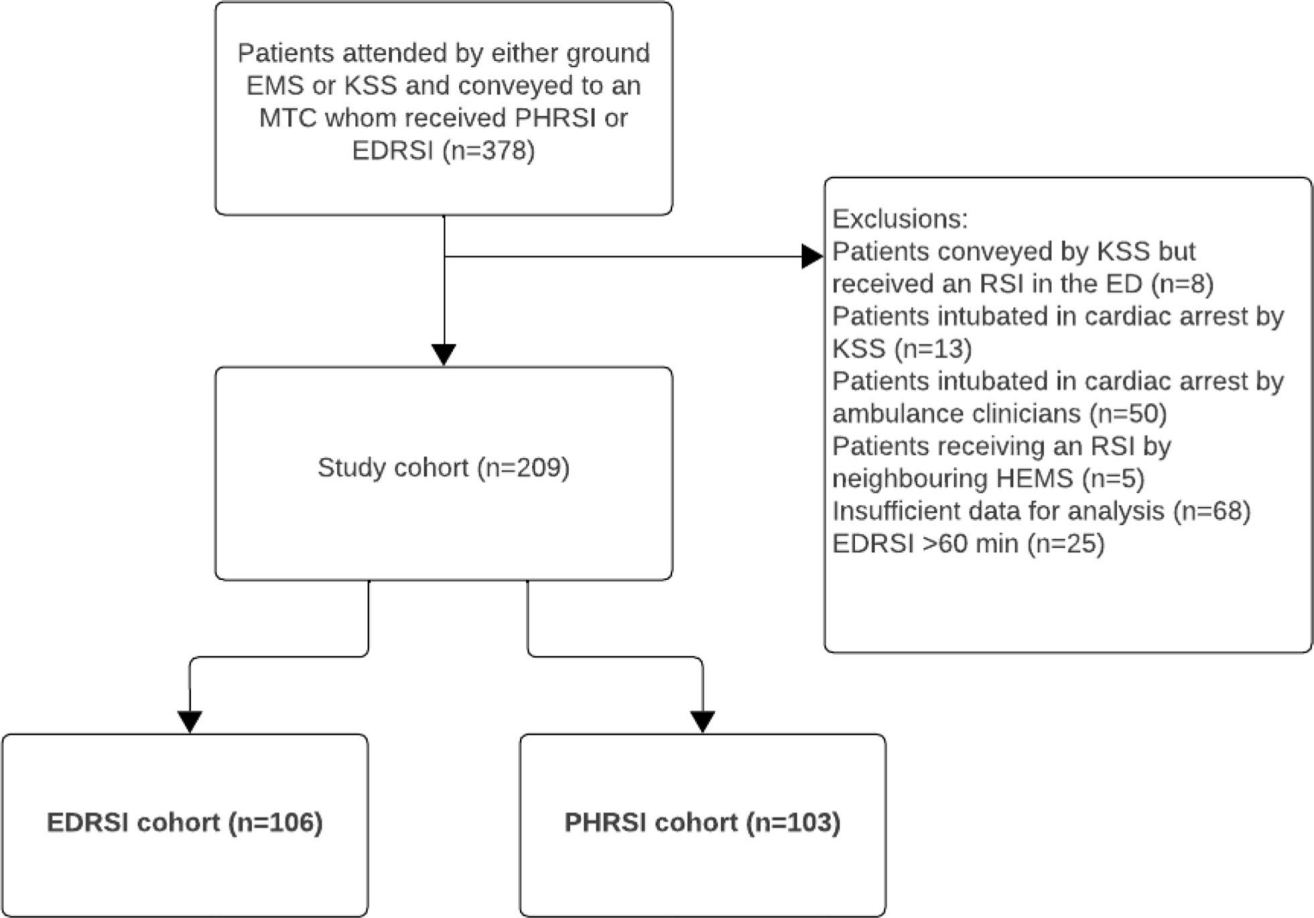
Derivation of the study population for EDRSI and PHRSI groups. EMS, Emergency Medical Service; KSS, Air Ambulance Charity Kent Surrey Sussex; MTC, Major Trauma Centre; RSI, Rapid Sequence Intubation; PHRSI, Pre-hospital Rapid Sequence Intubation; EDRSI, Emergency Department Rapid Sequence Intubation; ED, Emergency Department; HEMS, Helicopter Emergency Medical Services

During the study period, 378 trauma patients were conveyed by KSS or ground EMS to a participating MTC, received an RSI, and were TARN eligible. 169 patients were excluded, as summarised in the STROBE flow chart (Fig. 1). Further results refer to the remaining 209 patients who met study eligibility and inclusion criteria.

### Baseline characteristics and mechanism of injury

Most patients were male (n = 171, 82%) with a median age of 48 [28–65] years. 106 patients received an EDRSI and 103 received a PHRSI. The most common aetiology was blunt trauma (n = 198, 94%) involving a vehicular incident (n = 86, 29%). Over half of all patients who received a PHRSI were conveyed by helicopter (n = 67, 65%). Head injuries were the most injured body region (n = 134, 64%), followed by chest injuries (n = 26, 12%). All EDRSI patients arrived by land ambulance (Table 1).

**Table 1:** Baseline characteristics, injury profile and outcome stratified by RSI location

	All patients (n = 209)	EDRSI (n = 106)	PHRSI (n = 103)	*p value*
*Demographics*				
Age (years)	48 [28–65]	48 [28–66]	48 [28–64]	.6
Male (n [%])	171 [82]	86 [81]	85 [83]	.8
*Mode of arrival*				
Ambulance (n [%])	142 [68]	106 [100]	36 [35]	< .001
Helicopter (n [%])	67 [32]	0 [0]	67 [65]	
*Mechanism descriptors*				
Blunt Mechanism (n [%])	198 [95]	97 [92]	101 [98]	.034
Penetrating Mechanism (n [%])	11 [5]	9 [8]	2 [2]	
*Mechanism category*				
Burn (n [%])	2 [1]	1 [1]	1 [1]	< .001
Crush Injury (n [%])	2 [1]	2 [2]	0 [0]	
Fall < 2 m (n [%])	22 [11]	14 [13]	8 [8]	
Fall > 2 m (n [%])	49 [23]	23 [22]	26 [25]	
Vehicle Incident Collision (n [%])	86 [29]	40 [38]	46 [45]	
Shooting or stabbing injury (n [%])	2 [1]	2 [2]	0 [0]	
Other (n [%])	10 [6]	2 [8]	2 [2]	
*Injury descriptors*				
ISS	25 [19–34]	25 [17–29]	26 [23–35]	.02
*ISS Band (n [%])*				
ISS > 15	177 [85]	85 [80]	92 [89]	.17
ISS 9–15	28 [13]	18 [17]	10 [10]	
ISS 1–8	4 [2]	3 [3]	1 [1]	
On-scene presenting GCS (median [IQR])	7 [4–11]	7 [4–12]	7 [4–10]	.55
AIS and body region injured*				
AIS Abdomen (n [%])	4 [2]	2 [2]	2 [2]	.96
AIS Chest (n [%])	26 [12]	14 [13]	12 [12]	
AIS Head (n [%])	134 [64]	67 [63]	67 [65]	
AIS Limbs (n [%])	7 [3]	3 [3]	4 [4]	
AIS Multiple (n [%])	22 [11]	10 [9]	12 [12]	
AIS Other (n [%])	8 [4]	5 [5]	3 [3]	
AIS Spine (n [%])	8 [4]	5 [5]	3 [3]	
*Outcome*				
Alive at 30-days (n [%])	132 [63]	69 [65]	63 [61]	.65
Hospital length of stay in days	10 [4–30]	9 [3–24]	12 [5–33]	.2
Critical Care length of stay in days	4 [1–12]	3 [1–9]	6 [2–14]	.02

Categorical data are reported as frequency (n) and percentage (%) and numerical data as median (Interquartile range, IQR)* RSI* Rapid Sequence Intubation,* PHRSI* Pre-hospital Rapid Sequence Intubation,* EDRSI* Emergency Department Rapid Sequence Intubation,* CT* Computer Tomography,* ISS* Injury Severity Score,* GCS* Glasgow Coma Score,* AIS* Abbreviated Injury Scale. *AIS is denoted by the highest scoring body region, where multiple body regions had equal scoring the AIS region was marked as multiple

### Location of RSI and time to RSI

**Fig. 2:**
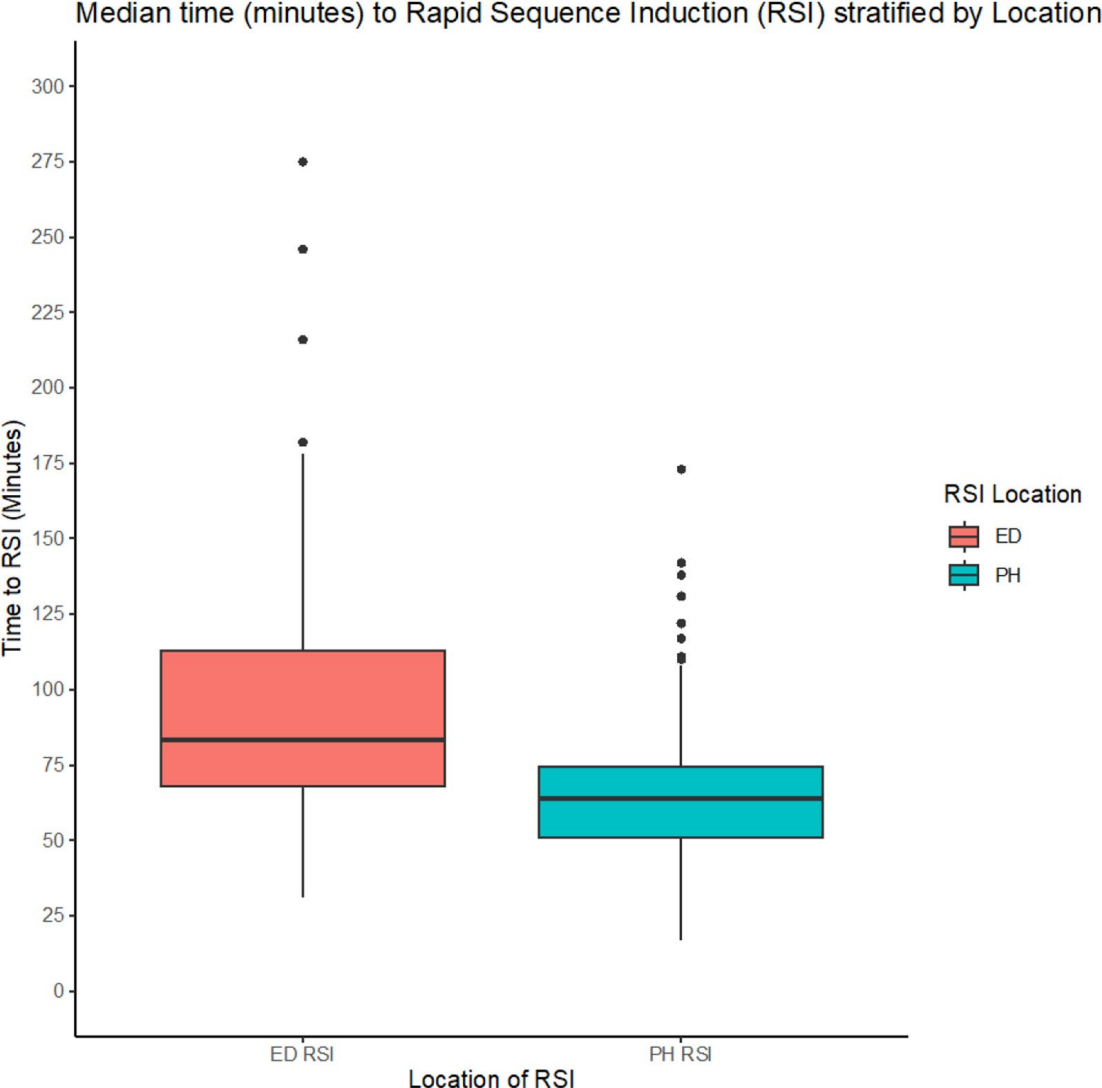
Median time to RSI (minutes) for intubation location stratified by RSI location. EDRSI, Emergency Department Rapid Sequence Intubation; PHRSI, Pre-hospital Rapid Sequence Intubation

**Fig. 3:**
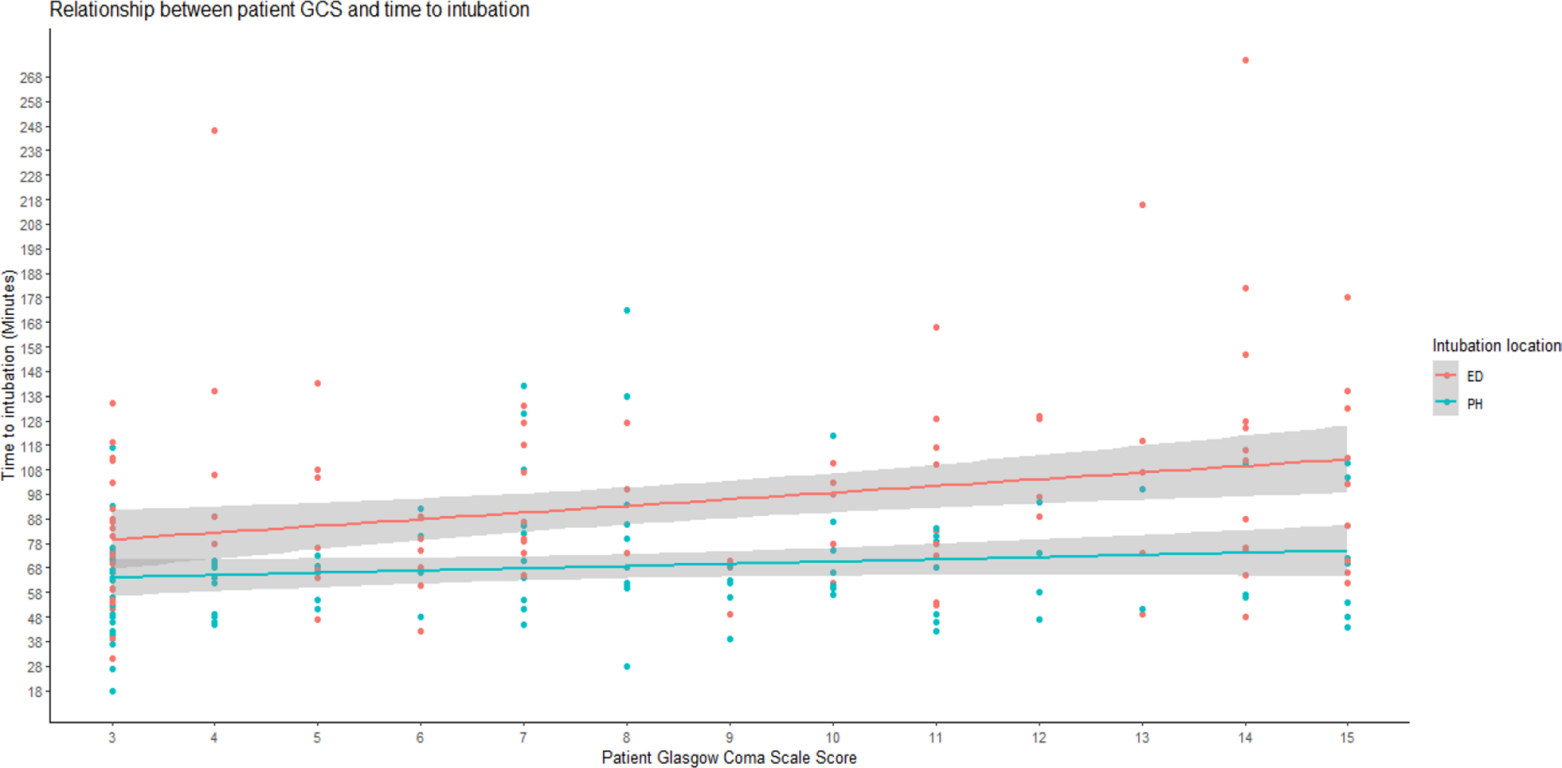
Relationship between GCS score and time to RSI (minutes). PH, Pre-hospital Rapid Sequence Intubation; ED, Emergency Department Rapid Sequence Intubation

Time to RSI from emergency call was faster in the PHRSI group (64 min [51–75] vs 84 min [68–113], *p* < 0.001) (Fig. 2). Linear regression was employed to test if the location of RSI (EDRSI or PHRSI) predicted overall time to RSI. The overall regression was statistically significant, and location of RSI significantly predicted time to RSI (Table 2). Further exploration of these findings demonstrated that other pre-hospital predictors influenced time to RSI including patient age. As patient age increased so did time to RSI (*p* < 0.001). Presenting GCS (as all components) was a predictor of time to RSI. As GCS increased, time to RSI also increased, with this effect being observed for both intubation locations (ED and PH). However, the relationship appeared more pronounced in the ED group, where higher GCS scores were associated with longer times to RSI compared to the PH group (Fig. 3).

**Table 2:** Results of linear regression analysis predicting time to RSI from either EDRSI or PHRSI

Variable	β	SE	t	95%CI
Location	57.33	9.47	6.05	−31.07 − 13.87
ISS	−0.30	0.19	−1.62	−0.68 − 0.06
GCS	2.26	0.53	4.24	1.21 – 3.31
Age	0.51	0.10	5.13	0.31 – 0.71

The adjusted R-squared for the model was 0.265, indicating that the model explains approximately 26.5% of the variance in intubation time after accounting for the number of predictors in the model. SE = standard error. β = standardized coefficient. t = t statistic. *Statistical significance at  *p* < 0.05.* CI* Confidence interval.* ISS* Injury severity score,* GCS* Glasgow coma score

### Post-admission anatomical and injury severity descriptors

The ISS and subsequent banded intervals are frequently used to categorise major (ISS > 15) and moderate (ISS 9–15) trauma. Most patients in our cohort were classified as suffering major trauma, (n = 177, 84%). The proportion of patients with an ISS > 15 was not significantly different between EDRSI and PHRSI groups (80% vs 89% respectively, *p* = 0.17). However, median ISS score was higher in the PHRSI group (ISS 26 [17–29] vs 25 [23–35], *p* = 0.02). The most injured body region, denoted by the abbreviated injury scale (AIS), in both groups was head (n = 134, 64%). Conversely to age and GCS, a lower ISS was associated with a longer time to RSI.

### Time and conveyance interval descriptors and analysis

Time intervals were explored to contextualise the differences between each stratified group. Time from first contact with an RSI capable team to delivery of RSI (HEMS scene arrival time to PHRSI or patient hospital arrival time to EDRSI) was significantly shorter in those having EDRSI (20-min, *p* < 0.001) (Table 2). Total pre-hospital time was also shorter in those receiving EDRSI (64-min, *p* < 0.001). Data were available from 169 patients (93/103 PHRSI and 76/106 EDRSI) to determine the travel time to hospital (conveyance interval). Median conveyance interval was 32 min [19–44], and the PHRSI group had a longer conveyance interval than the EDRSI group (40 [32–51 vs 18-min [12–29], *p* < 0.001). Finally, time from ED arrival to CT scan was 22 min shorter in the PHRSI cohort (21-min, *p* < 0.001) but overall time from 999/112 call to CT scan was 26 min shorter in the EDRSI group (Table 3).

**Table 3:** Time interval descriptors (minutes) for patients receiving a PHRSI or EDRSI

	All patients (n = 209)	EDRSI (n = 106)	PHRSI (n = 103)	*p* value
Pre-hospital time intervals				
Emergency call to RSI (min, median [IQR])	72 [57–98]	84 [68–113]	64 [51–75]	< .001
Arrival at destination hospital to RSI, or arrival of HEMS team to perform RSI (min, median [IQR])	22 [10–31]	18 [6–29]	25 [17–41]	< .001
Conveyance interval (min, median [IQR]	32 [19–44]	18 [12–29]	40 [32–51]	< .001
Total pre-hospital time (min, median [IQR])	98 [63–122]	64 [52–85]	115 [101–141]	< .001
In-hospital time intervals				
ED admission to CT scan (min, median [IQR]) *Missing (n)*	30 [18–45] *10*	43 [32–53] *6*	21 [15–30] *4*	< .001
Emergency call to CT scan (min, median [IQR])	119 [101–146]	107 [87–129]	132 [114–166]	< .001

Categorical data are reported as frequency (n) and percentage (%) and numerical data as median (Interquartile range, IQR) or mean (Standard Deviation, SD).* ED* Emergency Department,* CT* Computed Tomography,* RSI* Rapid Sequence Induction,* HEMS* Helicopter Emergency Medical Service. Total pre-hospital time, time from call to emergency services to arrival in hospital; Arrival of service to RSI, time from arrival of an RSI capable team to the patient until the patient undergoes RSI; Time from admission to CT scan, the time from arriving in ED to undergoing a CT scan

## Discussion

In this study, we report that the provision of emergency anaesthesia is expedited by a pre-hospital RSI-capable team across three trauma networks in the south of England. The prompt provision of emergency anaesthesia in patients with major trauma, who have lost the capacity to independently maintain their airway or ventilate effectively is critical [9].

On average, time from emergency call to RSI was 64-min in the PHRSI group, compared to 84-min in the EDRSI group, highlighting a significant timesaving for the delivery of this time-critical intervention. This time is comparable to other rural settings [9]. A national standard is important to enable comprehensive audit and evaluation, with the overarching goal of ensuring equitable healthcare provision. However, the applicability of a national standard in regions characterised by nuances in critical care delivery, trauma network capabilities and geographical factors warrants scrutiny. Time from first contact with an RSI-capable team to delivery of RSI was shorter in those receiving EDRSI. This is likely influenced by complexities seen in the pre-hospital environment that aren’t experienced in the ED. Entrapment and scene safety are two elements unique to the pre-hospital environment which could influence this parameter and prolong on-scene time.

National data indicate a 25% adherence to the prescribed 45-min window for RSI with a median time of 55 (45–70) minutes, [9] which is like our own data. Further, other Air Ambulance Services acknowledge difficulties in meeting this target [16]. In the undifferentiated trauma patient, RSI is often not the definitive intervention needed and therefore time to RSI should not be a stand-alone target, unless the patient has significant airway compromise or ventilatory failure.

The three trauma systems within which our study took place span a mixed rural and urban area covering 7390 km^2^ which introduces considerable variability in incident locality. Travel times are further influenced by factors such as weather conditions, mode of transportation (helicopter or ground vehicle) and other operational considerations. Pre-hospital times more than 60-min to non-tertiary centres are not uncommon and inevitably lead to a subsequent secondary transfer facilitated by ground EMS, thereby incurring further delay. Our study reports a significantly longer conveyance interval for those patients receiving a PHRSI signalling they were further from hospital demonstrating the unique operational utility behind HEMS.

Our study evidences a potential unmet demand in the provision of early advanced airway management for major trauma patients. In this cohort of major trauma patients, around 50% required urgent advanced airway management on arrival to ED within 60 min. In keeping with UK major trauma demography, those identified as having emergent airway interventions were mostly male and had suffered blunt trauma with associated head injury, and a high injury severity [4]. Those patients who had an extended time to RSI were likely to be older and present with a higher GCS score, which on detailed interrogation is the case for the outliers seen in the PHRSI group. Local pre-hospital service provision models with variable case mix may benefit from geo-temporal analysis, to further understand access to pre-hospital RSI following major traumatic injury.

The evidence for the benefit of pre-hospital advanced interventions, including RSI, which prolong on-scene times, has been mixed and is debated [25]. A historic tenet of trauma care has been the ‘golden hour’, the first hour after injury where initial resuscitation and expedient transport are thought to have the most impact on patient outcome. Resultantly, time has been thought of as a predominant factor determining outcomes after trauma [25]. However, a 2015 systematic review of level III evidence by Harmsen, et al. [25] showed that in an undifferentiated trauma patient cohort, there may be a positive correlation between on-scene time and survival, as well as total pre-hospital time and survival. This was except for patients suffering traumatic brain injury and those who were hypotensive after penetrating trauma. Our study found no significant difference in mortality between EDRSI and PHRSI groups, however the latter group had significantly longer total pre-hospital times. These data likely reflect the advancements of pre-hospital care since the advent of the ‘golden hour’ and suggest that there is a cohort of patients who benefit from advanced pre-hospital interventions. However, the NAP-4 study provided warning of the disproportionately high reported rates of airway complications outside of operating theatres. Despite the time-saving advantage, the decision to undertake PHRSI should, therefore, be made after careful consideration of the additional pre-hospital related risks of adverse airway related events, compared with the benefits associated with a definitive airway and titratable ventilation. The additional time and resources that PHRSI requires should be considered case-by-case, particularly in circumstances with multiple high acuity patients.

Injury Severity Score was significantly higher in those receiving PHRSI, demonstrating the larger burden of disease in this group and this is comparable to previous studies [17]. However, there was no difference in mortality between our two groups, contrary to an expected increased mortality in patients with higher injury burden. The only identified randomised control trial comparing mortality after PHRSI and EDRSI also found no significant difference in mortality [18]. Observational studies have shown that even after adjusting for ISS and GCS score, there is no significant difference in mortality for those receiving PHRSI versus EDRSI [19]. Crewdson et al. (2017) suggest it is probable that provision of increasingly enhanced pre-hospital care, results in a higher proportion of critically ill patients reach hospital alive rather than having resuscitation efforts terminated in the pre-hospital setting [20]. This could be reflected by our finding of significantly longer length of critical care admission in those receiving PHRSI. Our study findings are encouraging and support the potential benefit of early, definitive airway management on-scene.

Overall time from call to emergency services to CT scan was similar in our cohort and a study by Haslam, et al. [10] who also found that the median time from initial call to CT scan was 120 min. Time from call to emergency services to CT scan was found to be slower in the PHRSI group compared to EDRSI. Time to CT scan might be considered a surrogate for time to treatment, which suggests patients who receive an PHRSI are waiting longer to receive definitive care. However, it has been previously recognised that patients attended by HEMS teams are frequently further away from hospital than those seen by ground ambulance clinicians [27]. This is a principal factor in determining whether a patient will be flown by HEMS or conveyed by ground ambulance [27] and would account for the extended conveyance intervals and time from emergency call to CT scan seen in our cohort. Incident location for EDRSI patients is not collected by TARN, therefore determining the mean distance from hospital to further contextualise the conveyance interval for each group, was not possible. Centralising major trauma care has been a primary aim of trauma networks in England and has been shown to improve trauma outcomes [2]. However, a continuing criticism is that patients suffering major trauma may need to be transported over longer distances to an MTC, often bypassing nearby hospitals. A pre-hospital RSI capable team thereby forms an important component in facilitating centralised care, by supporting the extra distance major trauma patients need to travel to receive specialist, centralised care.

Our regression analysis found that higher presenting GCS was associated with longer time to RSI. GCS is easy to use and reproducible, however it has been previously shown that severe TBI (defined as AIS head ≥ 3) is poorly predicted by GCS alone, with more than one-fifth of TBI patients presenting with GCS > 8 [21]. Head injuries in patients presenting with high initial GCS may be occult and difficult to recognise, contributing to the increase in time to RSI. Indication for RSI may only become apparent after subsequent decompensation of a head injured patient. Increasing age was found to increase the time to RSI in our study. It has been recognised previously that older trauma patients are often under-triaged in pre-hospital care and trauma triage tools are inadequate in accurately identifying the older trauma patient [22]. False reassurance from low-energy mechanisms are a contributor to this bias. As the average age of our population continues to rise, so too will the burden of older trauma on trauma systems. The relationship between age and time to RSI found in our study provides further justification for ongoing research into improving pre-hospital care and outcomes for the older trauma patient.

Inherent limitations of the study design mean that confounding factors cannot be removed. A proportion of patients who received EDRSI had time of arrival in ED stated as identical to their RSI time. There is certainly a cohort of patients who require emergent interventions upon arrival to the ED and prior to handover which could explain some of these cases. However, identical timings in this instance could represent information bias in the form of retrospective or absent data entry from receiving teams in the ED. Similarly, a large proportion of the initial sample were excluded. In addition, limitations in the dataset meant that regression analysis on additional variables could not be undertaken i.e. it is unclear what proportion of patients who had ‘urgent’ tracheal intubation in ED (within 60 min) could have been delayed or may have not met PHRSI criteria. However, a large proportion who had a presenting GCS ≤ 8 on hospital arrival which confirms that many were emergent. Investigation of individual encounters was undertaken in those patients for whom time intervals were prolonged and classed as outliers (Fig. 2). Of these patients, a majority were over 75 years old, suffered head injury and presented to ED with a GCS score ≥ 13. As mentioned above, we posit that presenting injuries in these patients may have been occult and not immediately obvious to presenting teams. The subsequent, delayed deterioration of intracranial injury and following RSI could explain the delayed time intervals of these patients.

Baseline characteristics in each cohort differed with regards to mechanism of injury and ISS, introducing selection bias to the analysis. This could be explained by HEMS dispatch criteria, which could withhold dispatch to less severely injured patients. Information on physiological parameters and adverse airway incidents of patients was not available for our cohort. This could also further explain time intervals, as resuscitation of a patient with severe shock could cause prolonged on-scene time. This is an important factor in determining the safety and effectiveness of delivering PHRSI, as increased rates of adverse events could change decision making in patients who are particularly sensitive to secondary insults, such as traumatic brain injury. The study population spanned across a mixed urban and rural area in the South-East of England, containing three trauma networks and three MTCs. Generalisability of our results to aeromedical services in different parts of the UK and worldwide may be limited by the relative density of specialist MTCs across our region. Widespread data collection across the UK may help to increase the generalisability of our results.

## Conclusion

Major trauma patients requiring emergency anaesthesia received the intervention sooner following injury when performed in the pre-hospital setting by HEMS compared to those who were conveyed to a specialist hospital and RSI performed in-hospital. We demonstrated no difference in mortality between these two groups. The possible risks and benefits of earlier RSI to patients should be explored.

## Data Availability

The datasets used and/or analysed during the current study are available from the corresponding author on reasonable request.

## Abbreviations

HEMS: Helicopter emergency medical services
ED: Emergency department
PHRSI: Pre-hospital rapid sequence intubation
EDRSI: Emergency department rapid sequence intubation
TARN: Trauma audit research network
CT: Computed tomography
NCEPOD: National confidential enquiry into patient outcome and death
TBI: Traumatic brain injury
UK: United Kingdom
NICE: National institute for health and care excellence
MTC: Major trauma centre
EMS: Emergency medical services
KSS: Air ambulance charity kent surrey sussex
SOP: Standard operating procedure
NIHR: National institute of healthcare research
STROBE: Strengthening the reporting of observational studies in epidemiology
AIS: Abbreviated injury score
GCS: Glasgow coma scale
ISS: Injury severity score
SD: Standard deviation
IQR: Interquartile range
RECORD: Reporting of studies conducted using observational routinely collected data
